# *Quillaja saponaria* bark saponin protects Wistar rats against ferrous sulphate-induced oxidative and inflammatory liver damage

**DOI:** 10.1080/13880209.2017.1345950

**Published:** 2017-07-20

**Authors:** Mustafa Ahmed Abdel-Reheim, Basim Anwar Shehata Messiha, Ali Ahmed Abo-Saif

**Affiliations:** a Department of Pharmacology and Toxicology, Faculty of Pharmacy, Beni-Suef University, Beni Suef, Egypt;; b Department of Pharmacology and Toxicology, Faculty of Pharmacy, Nahda University, Beni Suef, Egypt

**Keywords:** Hepatotoxicity, *N*-acetylcysteine, nitric oxide synthase

## Abstract

**Context:** Saponins from different sources are historically reported in Chinese medicine to possess many beneficial effects. However, insufficient experimental data are available regarding the hepatoprotective potential of *Quillaja* bark saponin.

**Objective:** The protective effect of *Quillaja saponaria* Molina (Quillajaceae) bark triterpenoid saponin against iron-induced hepatotoxicity is compared to the standard *N*-acetylcysteine in adult male Wistar rats.

**Materials and methods:** Animals were divided into (six) groups, namely a normal control, an *N*-acetylcysteine control (300 mg/kg/day, p.o., 10 days), a saponin control (100 mg/kg/day, p.o., for 10 days), a hepatotoxicity control (two doses of ferrous sulphate, 30 mg/kg/day each, i.p., on 9th and 10th day), an *N*-acetylcysteine plus ferrous sulphate (standard treatment) and a saponin plus ferrous sulphate (test treatment) group. Hepatocyte integrity loss markers (serum ALT, AST, ALP, GGT and LDH), oxidative stress markers (hepatic MDA, GSH and NO_x_), dyslipidaemic markers (serum TC and TG) and hepatocyte functioning markers (serum bilirubin and albumin) were assessed.

**Results:***Quillaja* bark saponin decreased iron-induced elevation of ALT (reaching 57% of hepatotoxicity control), AST (66%), ALP (76%), GGT (60%), LDH (54%), MDA (65%), NO_x_ (77%), TC (70%), TG (54%), and total (54%), direct (54%) and indirect (54%) bilirubin, coupled with increased GSH (219%) and albumin (159%) levels. Histopathological study strongly supported biochemical estimations, while immunohistochemical study showed marked effect on eNOS and iNOS expression.

**Conclusions:***Quillaja* bark saponin has a good hepatoprotective effect. Amelioration of oxidative stress and suppression of NOS expression, with resultant maintenance of hepatocyte integrity and functioning, may explain this beneficial effect.

## Introduction

The liver is the largest internal organ in the human body and is localized in a strategic position in the abdominal cavity between the digestive tract, the spleen and the general circulation (Kudo 2000; Vekemans and Braet 2005). Being the main detoxifying organ in the body, it is highly susceptible to injury with chemotherapeutic agents, drugs, environmental toxins and biological pathogens (Stephens et al. 2014).

Iron is an important trace element in the body, being found in functional form in haemoglobin, myoglobin and cytochrome enzymes (Reddy and Lokesh 1996). However, iron overload is known to be associated with oxidative stress-induced disorders including hepatocellular necrosis and cirrhosis (Kohgo et al. 2008; Sawada et al. 2015). Being a transition metal, it is a potent catalyst to free radical generation reactions like Fenton and Haber–Weiss reactions (Halliwell and Gutteridge 1990, 1992; Dixon and Stockwell 2014). Additionally, Bantu siderosis is an attractive example for the harmful effects of iron overload (Senba et al. 1989). Based on these data, hepatotoxicity was induced in the current study using an iron overload model in a simulation to such clinical situations.

Saponins are amphipathic foaming glycosides characterized by having hydrophilic glycoside moieties combined with a lipophilic aglycone (Osbourn et al. 2011). In plants, saponins perform many important digestive (Cheeke 2000), antibacterial (Avato et al. 2006), antifungal (Wink 1988) and other functions. Saponins were reported to possess many beneficial effects clinically as well. It was also found that tea-leaf saponins antagonize the action of leukotriene D_4_, one of the chemical mediators of inflammatory reactions (Sagesaka et al. 1996). Saponin-containing extracts like marshmallow were reported to possess anti-ulcer effects in different animal models (Zaghlool et al. 2015a, 2015b). Extensive studies have also been carried out on the hypocholesterolaemic, immunostimulant and anticarcinogenic properties of saponins (Francis et al. 2002). Interestingly, saponins were reported to possess beneficial anticancer effects based on their amphiphilic nature enabling such compounds to cause destruction of tumour cell membranes. Such application is limited by their ability to cause haemolysis of blood cells *in vitro* (Lorent et al. 2014; Top et al. 2017). However, not all saponins are alike regarding such haemolytic activity. Previous researchers reported great variations among saponins regarding haemolytic activity based on their polarity and chemistry of their aglycones and sapogenins (Top et al. 2017; Vo et al. 2017). For instance, the triterpenoid saponin obtained from *Quillaja saponaria* Molina (Quillajaceae) bark was reported to be relatively non-haemolytic and was approved as an immunostimulant adjuvant in marketed vaccines (de Groot and Müller-Goymann 2016).

Saponins obtained from different extracts were reported to possess beneficial hepatoprotective effects. However, no sufficient data are available regarding the hepatoprotective potential of *Quillaja* bark saponin against experimental liver injury. *Quillaja saponaria* bark saponin is a triterpenoid saponin complex in which the sapogenin (aglycone) is a triterpene termed quillaic acid (Guo et al. 1998; Guo and Kenne 2000). Total saponin content of the plant extract may exceed 100 members, most abundantly Quil-A (So et al. 1997; Barr et al. 1998). *Quillaja* bark saponin is characteristically different from other triterpenoid plant saponins regarding chemical structure. The difference may be represented by a fatty acid domain and a triterpene aldehyde group at carbon 4 of the triterpene (Kensil et al. 1995).

Accordingly, the present investigation aimed to elucidate the possible protective effect of saponin from *Quillaja saponaria* bark against iron-induced hepatotoxicity as compared to *N*-acetylcysteine as a standard treatment in adult male Wistar rats. No previous study reported the hepatoprotective effect of *Quillaja* bark saponin against iron-induced liver injury experimentally. In this study, hepatic integrity loss markers were measured, including serum alanine aminotransferase (ALT), serum aspartate aminotransferase (AST), serum alkaline phosphatase (ALP), serum γ-glutamyltransferase (GGT), serum lactate dehydrogenase (LDH), serum albumin and serum bilirubin. Dyslipidaemic markers were also measured, including serum total cholesterol (TC) and serum triglycerides (TG), in addition to oxidative/nitrosative stress markers including reduced glutathione (GSH) stores, malondialdehyde (MDA) content and nitrate/nitrite (NO_x_) production. Additionally, histopathological and immunohistochemical studies were performed to assess histopathological lesions in addition to tissue endothelial nitric oxide synthase (eNOS) and inducible nitric oxide synthase (iNOS).

## Materials and methods

### Animals

Experiments were performed using male Wistar rats obtained from the National Research Center (Cairo, Egypt). Rats weighing 200–250 g were used for the experiments. Animals were housed in plastic cages (28 cm × 43 cm ×18 cm) and were maintained under conventional laboratory conditions throughout the study. They were fed standard pellet chow (El-Nasr Chemical Co., Cairo, Egypt) and were allowed food and water *ad libitum*. The number of rats in each group was initially 10 rats with minor loss due to mortality and experimentation errors. Our work was approved by the ‘Experimental Animal Ethics Committee, Beni-Suef University’. Animal handling and experimental procedures were conducted according to the ‘Rules and Guidelines of the Animal House of Beni-Suef University, section B’ approved by the ‘Pharmacology and Toxicology Department’ in 2009. These followed the guidelines of the National Institutes of Health (NIH) Guide for Care and Use of Laboratory Animals (Publication No. 85–23, revised 1985).

### Drugs

#### *N*-Acetylcysteine

*N*-Acetylcysteine was purchased from Sigma-Aldrich (St. Louis, MO) and orally administered in a dose of 300 mg/kg/day (Jones 1998; Heidari et al. 2014).

#### Saponin

Saponin from *Quillaja* bark (triterpenoid quillaic acid saponin) was purchased from Sigma-Aldrich (St. Louis, MO) and orally administered in a dose of 100 mg/kg/day (Jeong et al. 1997; Huang et al. 2012).

#### Chemicals and kits

Serum ALT, AST, albumin and bilirubin reagent kits were obtained from Diamond Diagnostics, Cairo, Egypt. ALP kits were obtained from Biodiagnostics, Cairo, Egypt. Disodium hydrogen phosphate and orthophosphoric acid were obtained from Merck (Darmstadt, Germany). Ellman’s reagent, ferrous sulphate, reduced glutathione (GSH), malondialdehyde (MDA), *N*-(1-naphthyl) ethylenediamine dihydrochloride (NEDD), sulphanilamide, sulphosalicylic acid, thiobarbituric acid and vanadium trichloride were obtained from Sigma-Aldrich (St. Louis, MO). The eNOS and iNOS primary antibodies were obtained from Proteintech (Rosemont, IL). GGT kits were obtained from Analyticon (Lichtenfels, Germany). LDH kits were obtained from Biosystems (Barcelona, Spain). TC and TG assay kits were obtained from Spinreact (Barcelona, Spain). All other chemicals used were of the analytical grade or equal quality.

#### Experimental design

Animals were randomized into six groups, each of 8–10 rats. Group 1 was kept as a normal control group. Group 2 was kept as an *N*-acetylcysteine control group. Group 3 was kept as a saponin control group. Group 4 was kept as a hepatotoxicity control group, receiving ferrous sulphate only. Group 5 was kept as a standard treatment group receiving *N*-acetylcysteine in addition to ferrous sulphate. Group 6 was the test treatment group, receiving saponin plus ferrous sulphate. Test agents or vehicles were administered orally on a daily basis for 10 consecutive days. Hepatic injury was induced by two i.p. injections of ferrous sulphate (30 mg/kg), given on 9th and 10th day.

#### Induction of liver injury

The model was modified from the method described by Reddy and Lokesh (1996) and Bhattacharya et al. (2000a). Hepatic injury was induced by i.p. injection of two doses of ferrous sulphate (30 mg/kg each) on 9th and 10th day. Animals were fasted for 18 h before receiving the last protective dose on day 10. One hour after the last drug dose, the second oral dose of ferrous sulphate was administered. Animals were then fasted for an additional 1 h. Animals were anesthetized with thiopental sodium (75 mg/kg, i.p.) and blood samples were collected from retro-orbital plexus using heparinized microcapillary tubes. Rats were then sacrificed by cervical dislocation to separate liver samples (Kiran et al. 2012).

### Manipulation of samples

#### Blood samples

After collecting blood samples in centrifuge tubes, the tubes were allowed to coagulate at room temperature, placed in water bath at 37 °C for 10 min and then centrifuged at 1000 *g* for 20 min. The clear serum was separated and was used for analysis of biochemical parameters, including ALT, AST, ALP, LDH, GGT, bilirubin, albumin, TC and TG.

#### Liver samples

Soon after sacrificing, the abdominal cavities were opened and livers were carefully separated, washed with ice-cold saline and the median and left hepatic lobes were separated. The liver lobes were used for the preparation of liver homogenate and of sections for histopathological and immunohistochemical examination.

#### Preparation of liver homogenate

To prepare 20% liver homogenate, 1 g of the median lobe was homogenized with 5 volumes of isotonic ice-cooled normal saline using a homogenizer (IKA homogenizer, Model T 25 digital ULTRA-TURRAX, Staufen, Germany) for estimation of hepatic MDA, GSH and NO_x_ levels.

#### Preparation of slides for histopathological and immunohistochemical examination

A portion of each liver was kept in well-sealed containers in formalin solution in normal saline (10%) prior to wax embedding, sectioning and staining.

### Measurement of biomarkers

#### Determination of serum biomarkers

Serum biomarkers were estimated using commercial kits based on the principles described earlier. Serum ALT and AST were determined according to the method of Reitman and Frankel (1957). Serum ALP was determined according to the method of Belfield and Goldberg (1970). Serum GGT was determined according to the method of Szasz (1976). Serum LDH activity was determined according to the method of Vassault (1983). Serum TG level was assayed according to the method described by Bucolo and David (1973). Serum TC level was assayed according to the method described by Boussekine et al. (2014). Serum albumin was determined according to the method of Tietz (1990). Serum total bilirubin was determined according to the method of Tietz (1995). Serum direct bilirubin was determined according to the method of Tietz (1995). Indirect bilirubin can be obtained from subtracting direct bilirubin from total bilirubin according to the method of Tietz (1995).

#### Estimation of hepatic biomarkers

Hepatic GSH was measured in liver homogenate according to the method described by Sedlak and Lindsay (1968). Lipid peroxidation was determined in liver homogenate as thiobarbituric acid reactive substances (TBARS) measured as malondialdehyde (MDA) according to the method of Uchiyama and Mihara (1978). Hepatic NO_x_ production in liver tissue was assayed according to the method described by Miranda et al. (2001).

#### Histopathological assessment of liver injury

Samples were taken from the livers of rats in different groups and fixed in 10% formalin solution in normal saline for 24 h. Washing was done in tap water and then serial dilutions of alcohol (methyl, ethyl and absolute ethyl) were used for dehydration. Specimens were cleared in xylene and embedded in paraffin at 56 °C in hot air oven for 24 h. Paraffin bees wax tissue blocks were prepared for sectioning at 4 microns thickness by sledge microtome. The obtained tissue sections were collected on glass slides, deparaffinized and stained with haematoxylin and eosin (H&E) stain for routine examination. Then, examination was done using a light microscope attached to a digital camera.

#### Immunohistochemistry

Immunohistochemical study was performed according to the method of Merz et al. (1995). The labelled streptavidin–biotin (LSAB) staining method was used in which horseradish peroxidase (HRP), streptavidin and two-tiered antibodies are employed to reveal the presence of antigens in a variety of tissues and cell preparations. After the primary antibody has been bound to a target antigen, namely iNOS and eNOS, a secondary antibody that binds specifically to that primary antibody was used. HRP-labelled streptavidin is then bound to the biotinylated secondary antibody and the entire complex is revealed by adding a substrate/chromogen mixture which creates an intense colour deposit through the activity of the bound enzyme.

#### Statistical analysis

Data were expressed as means of 6–8 values ± standard error of the means (SEM). Comparison between different treatments was carried out using analysis of variance (ANOVA) test followed by Tukey–Kramer multiple comparisons test. Mean values were examined using ‘mean validity test’ prior to ANOVA test, where a valid mean is at least 10 times its standard error and 2.5 times its standard deviation. Differences were considered statistically significant at *p* < 0.05. Statistical analysis was done by the aid of GraphPad prism and GraphPad instant computer software (San Diego, CA). Superscript letters were assigned to the mean values of measurements referring to the groups within which significant differences exist. The letter (a) was denoted to mean values significantly different from those of the normal control group, the letter (b) was assigned to mean values significantly different from those of the hepatotoxicity control group, and the letter (c) denoted significant difference compared with the respective *N*-acetylcysteine treatment group mean value.

## Results

### Effect of 10 days daily oral administration of *N*-acetylcysteine and saponin on hepatocyte integrity loss markers in normal rats

**Figure 1.**
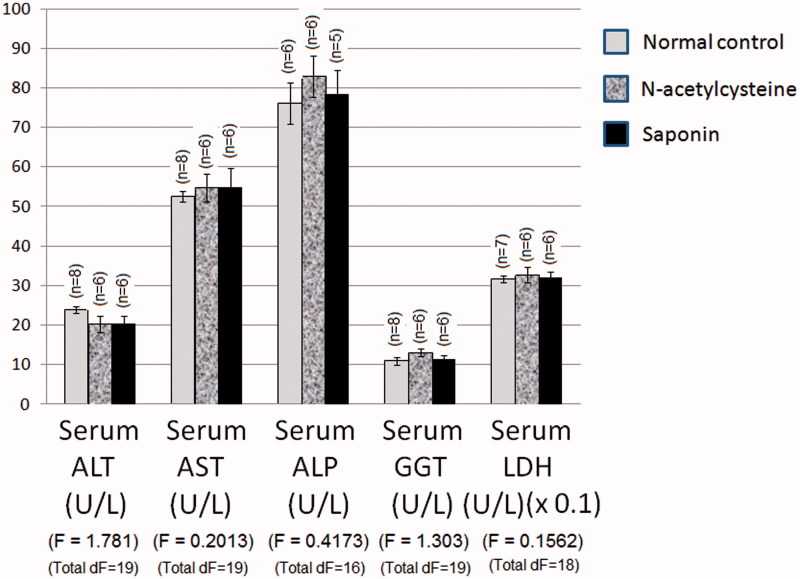
Effect of 10 days daily oral administration of *N*-acetylcysteine and saponin on hepatocyte integrity loss markers in normal rats.

Normal control values of serum ALT and AST activities were 23.9 and 52.5 U/L, respectively (Figure 1). *N*-acetylcysteine and saponin alone did not significantly affect serum ALT or AST activities of rats as compared to normal values. The normal control values of serum ALP, GGT and LDH activities of normal rats were 76.1, 10.9 and 317.2 U/L, respectively, which were not significantly affected by *N*-acetylcysteine or saponin administration alone.

### Effect of 10 days daily oral administration of *N*-acetylcysteine and saponin on hepatocyte integrity loss markers in rats with ferrous sulphate-induced hepatotoxicity

Values of serum ALT and AST activities of ferrous sulphate-intoxicated rats were 60.8 and 124.8 U/L, respectively, which were significantly higher than the respective normal control values (Table 1). Pre-treatment with *N*-acetylcysteine and saponin significantly reduced ferrous sulphate-induced elevation in serum ALT activity to 31.4 and 34.4 U/L, respectively. Pre-treatment with *N*-acetylcysteine and saponin significantly reduced ferrous sulphate-induced elevation in serum AST activity to 73.4 and 82.8 U/L, respectively.

**Table 1.** Effect of 10 days daily oral administration of *N*-acetylcysteine and saponin on hepatocyte integrity loss markers in rats with ferrous sulphate-induced hepatotoxicity.

Parameters	ALT (U/L)	AST (U/L)	ALP (U/L)	GGT (U/L)	LDH (U/L)
Statistical data	(F = 74.24, dF between groups =3, dF within groups =27)	(F = 146.80, dF between groups =3, dF within groups =28)	(F = 89.86, dF between groups =3, dF within groups =22)	(F = 63.59, dF between groups =3, dF within groups =28)	(F = 97.36, dF between groups =3, dF within groups =23)
Normal control (2% Tween 80, p.o.)	23.9 ± 0.80 (*n* = 8)	52.5 ± 1.37 (*n* = 8)	76.1 ± 5.27 (*n* = 6)	10.9 ± 0.97 (*n* = 8)	317.2 ± 8.85 (*n* = 7)
Hepatotoxicity control (FeSO_4_, 30 mg/kg for 2 days, i.p.)	60.8 ± 2.38^a^ (*n* = 8)	124.8 ± 3.24^a^ (*n* = 8)	351.5 ± 10.67^a^ (*n* = 7)	58.8 ± 3.75^a^ (*n* = 8)	1098.0 ± 47.53^a^ (*n* = 8)
*N*-acetylcysteine + Ferrous sulphate	31.4 ± 1.91^ab^ (*n* = 8)	73.4 ± 2.91^ab^ (*n* = 8)	162.5 ± 14.30^ab^ (*n* = 7)	29.4 ± 2.26^ab^ (*n* = 8)	561.7 ± 33.90^ab^ (*n* = 6)
Saponin + Ferrous sulphate	34.4 ± 2.15^ab^ (*n* = 7)	82.8 ± 2.10^ab^ (*n* = 8)	267.9 ± 17.12^abc^ (*n* = 6)	35.3 ± 2.11^ab^ (*n* = 8)	598.1 ± 30.27^ab^ (*n* = 6)

^a^Significantly different from normal control group at *p* < 0.05.

^b^Significantly different from hepatotoxicity control group at *p* < 0.05.

^c^Significantly different from *N*-acetylcysteine control group at *p* < 0.05.

The values of serum ALP, GGT and LDH activities of ferrous sulphate control rats were 351.5, 58.8 and 1098.0 U/L, respectively, which were significantly higher than the normal control values. Pretreatment with *N*-acetylcysteine and saponin significantly reduced ferrous sulphate-induced elevation in serum ALP activity to 162.5 and 267.9 U/L, respectively. Pretreatment with *N*-acetylcysteine and saponin significantly reduced ferrous sulphate-induced elevation in serum GGT activity to 29.4 and 35.3 U/L, respectively. Pretreatment with *N*-acetylcysteine and saponin significantly reduced ferrous sulphate-induced elevation in serum LDH activity to 561.7 and 598.1 U/L, respectively.

### Effect of 10 days daily oral administration of *N*-acetylcysteine and saponin on nitro-oxidative stress markers in normal rats

**Figure 2.**
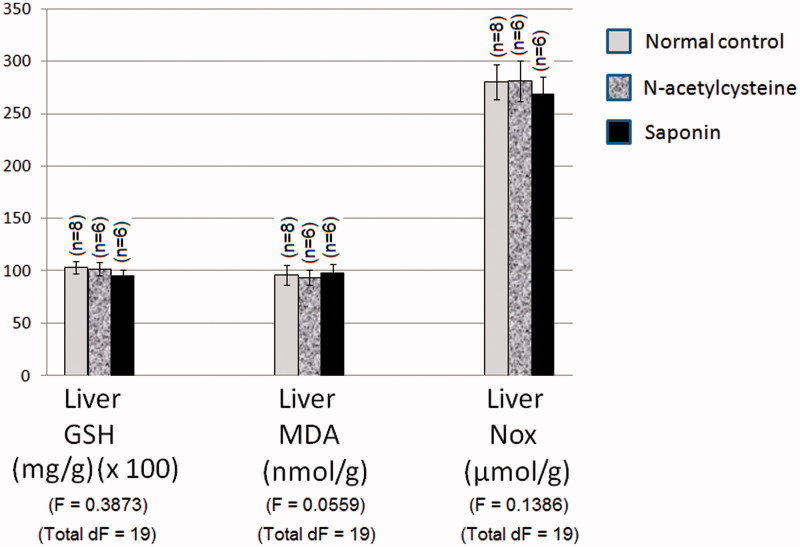
Effect of 10 days daily oral administration of *N*-acetylcysteine and saponin on nitro-oxidative stress markers in normal rats.

Normal control values of liver GSH content, MDA content and NO_x_ production were 1.03 mg/g wet tissue, 96.09 nmol/g wet tissue and 280.00 μmol/g wet tissue, respectively. *N*-Acetylcysteine and saponin alone did not significantly affect liver GSH content, MDA content and NO_x_ production in rats as compared to normal values (Figure 2).

### Effect of 10 days daily oral administration of *N*-acetylcysteine and saponin on nitro-oxidative stress markers in rats with ferrous sulphate-induced hepatotoxicity

Values of liver GSH content, MDA content and NO_x_ production of ferrous sulphate control rats were 0.31 mg/g wet tissue, 230.50 nmol/g wet tissue and 589.50 μmol/g wet tissue, respectively, which were significantly different from the respective normal group values (Table 2). Pretreatment with *N*-acetylcysteine and saponin significantly corrected ferrous sulphate-induced suppression in liver GSH content reaching 0.83 and 0.68 mg/g wet tissue, respectively. Pretreatment with *N*-acetylcysteine and saponin significantly reduced ferrous sulphate-induced elevation in liver MDA content to reach 137.50 and 150.10 nmol/g wet tissue, respectively. Pretreatment with *N*-acetylcysteine and saponin significantly reduced ferrous sulphate-induced elevation in liver NO_x_ production to be 382.00 and 453.30 μmol/g wet tissue, respectively.

**Table 2.** Effect of 10 days daily oral administration of *N*-acetylcysteine and saponin on nitro-oxidative stress markers in rats with ferrous sulphate-induced hepatotoxicity.

Parameters	Liver GSH content (mg/g wet tissue)	Liver MDA content (nmol/g wet tissue)	Liver NO_x_ production (μmol/g wet tissue)
Statistical data	(F = 78.97, dF between groups = 3, dF within groups =28)	(F = 18.94, dF between groups = 3, dF within groups =28)	(F = 26.62, dF between groups = 3, dF within groups =28)
Normal control (2% Tween 80, p.o.)	1.03 ± 0.057 (*n* = 8)	96.09 ± 9.496 (*n* = 8)	280.00 ± 16.810 (*n* = 8)
Hepatotoxicity control (FeSO_4_, 30 mg/kg, i.p.)	0.31 ± 0.025^a^ (*n* = 8)	230.50 ± 17.480^a^ (*n* = 8)	589.50 ± 20.070^a^ (*n* = 8)
*N*-Acetylcysteine + Ferrous sulphate	0.83 ± 0.019^ab^ (*n* = 8)	137.50 ± 12.950^b^ (*n* = 8)	382.00 ± 24.350^ab^ (*n* = 8)
Saponin + Ferrous sulphate	0.68 ± 0.022^abc^ (*n* = 8)	150.10 ± 10.250^ab^ (*n* = 8)	453.30 ± 35.670^ab^ (*n* = 8)

### Effect of 10 days daily oral administration of *N*-acetylcysteine and saponin on dyslipidaemia markers in normal rats

**Figure 3.**
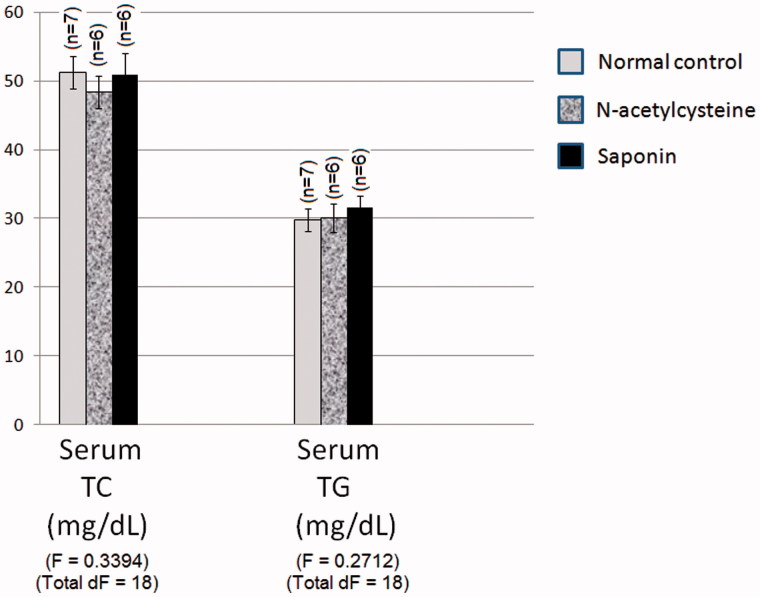
Effect of 10 days daily oral administration of *N*-acetylcysteine and saponin on dyslipidaemia markers in normal rats.

Normal group values of serum TC and TG levels of normal rats were 51.2 and 29.8 mg/dL, respectively. Pretreatment with *N*-acetylcysteine and saponin did not significantly affect serum TC and TG levels of rats as compared to normal values (Figure 3).

### Effect of 10 days daily oral administration of *N*-acetylcysteine and saponin on dyslipidaemia markers in rats with ferrous sulphate-induced hepatotoxicity

Values of serum TC and TG levels of ferrous sulphate control rats were 118.5 and 97.8 mg/dL, respectively, which were significantly higher than the normal control value (Table 3). Pretreatment with *N*-acetylcysteine and saponin significantly reduced ferrous sulphate-induced elevation in serum TC level to reach 75.8 and 83.2 mg/dL, respectively. Pretreatment with *N*-acetylcysteine and saponin significantly reduced ferrous sulphate-induced elevation in serum TG level to be 59.5 and 53.1 mg/dL, respectively.

**Table 3.** Effect of 10 days daily oral administration of *N*-acetylcysteine and saponin on dyslipidaemia markers in rats with ferrous sulphate-induced hepatotoxicity.

Parameters	Serum TC (mg/dL)	Serum TG (mg/dL)
Statistical data	(F = 83.85, dF between groups =3, dF within groups =24)	(F = 98.69, dF between groups =3, dF within groups =23)
Normal control (2% Tween 80, p.o.)	51.2 ± 2.31 (*n* = 7)	29.8 ± 1.62 (*n* = 7)
Hepatotoxicity control (FeSO_4_, 30 mg/kg, i.p.)	118.5 ± 3.81^a^ (*n* = 8)	97.8 ± 4.08^a^ (*n* = 7)
*N*-Acetylcysteine + Ferrous sulphate	75.8 ± 2.86^ab^ (*n* = 7)	59.5 ± 2.85^ab^ (*n* = 7)
Saponin + Ferrous sulphate	83.2 ± 3.11^ab^ (*n* = 6)	53.1 ± 2.20^ab^ (*n* = 6)

^a^Significantly different from normal control group at *p* < 0.05.

^b^Significantly different from hepatotoxicity control group at *p* < 0.05.

### Effect of 10 days daily oral administration of *N*-acetylcysteine and saponin on functional markers in normal rats

**Figure 4.**
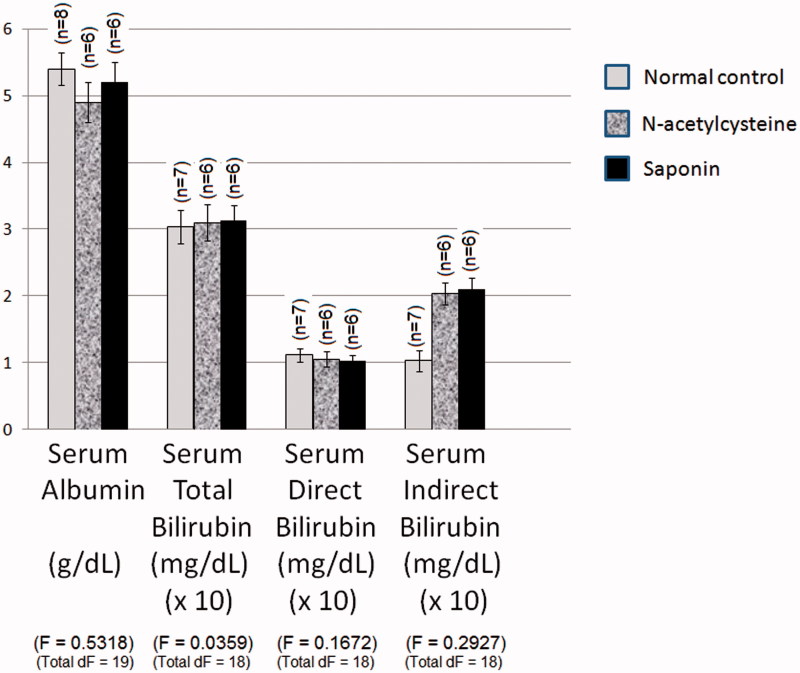
Effect of 10 days daily oral administration of *N*-acetylcysteine and saponin on functional markers in normal rats.

Normal group value of serum albumin level of normal rats was 5.349 g/dL (Figure 4). *N*-Acetylcysteine and saponin did not significantly affect serum albumin level of normal rats. The normal control values of total serum, direct and indirect bilirubin levels were 0.304, 0.111 and 0.193 mg/dL, respectively. *N*-Acetylcysteine and saponin did not significantly affect total serum, direct and indirect bilirubin levels of rats as compared to normal values.

### Effect of 10 days daily oral administration of *N*-acetylcysteine and saponin on functional markers in rats with ferrous sulphate-induced hepatotoxicity

Value of serum albumin level of ferrous sulphate control rats was 2.748 g/dL, which was significantly lower than the normal control value (Table 4). Pretreatment with *N*-acetylcysteine and saponin significantly inhibited ferrous sulphate-induced reduction in serum albumin level to be 4.538 and 4.367 g/dL, respectively.

**Table 4.** Effect of 10 days daily oral administration of *N*-acetylcysteine and saponin on functional markers in rats with ferrous sulphate-induced hepatotoxicity.

Parameters	Serum albumin (g/dL)	Serum total bilirubin (mg/dL)	Serum direct bilirubin (mg/dL)	Serum indirect bilirubin (mg/dL)
Statistical data	(F = 15.10, dF between groups = 3, dF within groups =26)	(F = 98.06, dF between groups = 3, dF within groups =24)	(F = 77.85, dF between groups = 3, dF within groups =24)	(F = 108.60, dF between groups =3, dF within groups =24)
Normal control (2% Tween 80, p.o.)	5.349 ± 0.2351 (*n* = 8)	0.304 ± 0.0251 (*n* = 7)	0.111 ± 0.0098 (*n* = 7)	0.193 ± 0.0156 (*n* = 7)
Hepatotoxicity control (FeSO_4_, 30 mg/kg, i.p.)	2.748 ± 0.2529^a^ (*n* = 8)	1.847 ± 0.0739^a^ (*n* = 7)	0.679 ± 0.0310^a^ (*n* = 7)	1.168 ± 0.0444^a^ (*n* = 7)
*N*-Acetylcysteine + Ferrous sulphate	4.538 ± 0.3603^b^ (*n* = 7)	0.842 ± 0.0478^ab^ (*n* = 7)	0.296 ± 0.0191^ab^ (*n* = 7)	0.546 ± 0.0291^ab^ (*n* = 7)
Saponin + Ferrous sulphate	4.367 ± 0.3151^b^ (*n* = 7)	1.000 ± 0.0911^ab^ (*n* = 7)	0.366 ± 0.0381^ab^ (*n* = 7)	0.634 ± 0.0541^ab^ (*n* = 7)

The values of serum total, direct and indirect bilirubin levels of ferrous sulphate control rats were 1.847, 0.679 and 1.168 mg/dL, respectively, which were significantly higher than the normal control values. Pretreatment with *N*-acetylcysteine and saponin significantly reduced ferrous sulphate-induced elevation in serum total bilirubin level to be 0.842 and 1.000 mg/dL, respectively. Pretreatment with *N*-acetylcysteine and saponin significantly inhibited ferrous sulphate-induced elevation in serum direct bilirubin level. Pretreatment with *N*-acetylcysteine and saponin significantly inhibited ferrous sulphate-induced elevation in serum indirect bilirubin.

### Effect of 10 days daily oral administration of *N*-acetylcysteine and saponin on liver histopathology in rats with ferrous sulphate-induced hepatotoxicity

**Figure 5.**
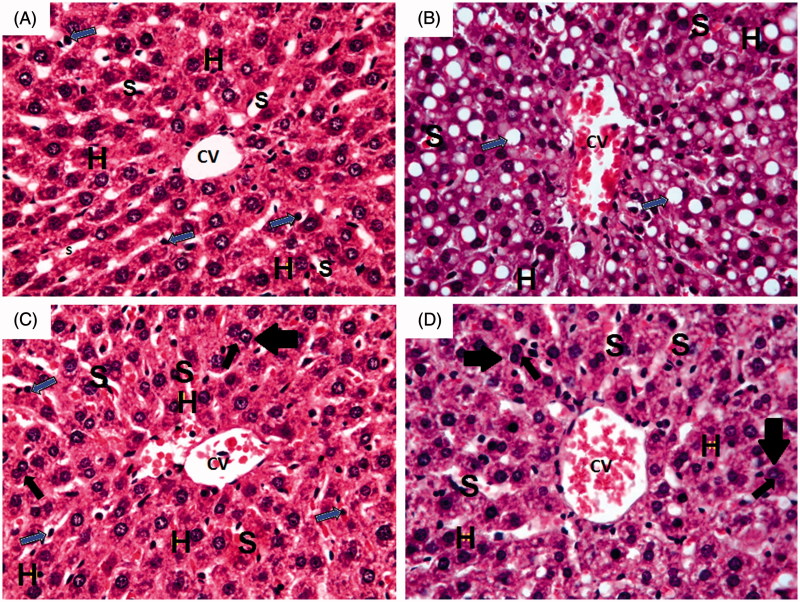
Photomicrographs of liver section obtained from different groups (H&E; 400×), where  (A) normal control group showing normal hepatic architecture with central vein (CV) and radiating cords of normal hepatocytes (H) with central rounded vesicular nuclei and prominent nucleoli. Hepatic cords are separated by blood sinusoids (S) lined with endothelium and von Kupffer cells (blue arrow); (B) ferrous sulphate group showing dilated congested central vein (CV) with congested blood sinusoids (S). Massive fatty infiltration of hepatocytes (H) with some hepatocytes acquired the signet ring appearance (blue arrow); (C) *N*-acetylcysteine plus ferrous sulphate group showing congested central vein (CV). Normal hepatocytes (H) are separated by slightly dilated congested blood sinusoids (S) with activated von Kupffer cells (blue arrow). Binucleated cells (black arrow) can be seen; (D) saponin plus ferrous sulphate group showing congested central vein (CV). Normal hepatocytes (H) are separated by slightly dilated congested blood sinusoids (S) with activated von Kupffer cells (blue arrow). Binucleated cells (black arrow) can be seen.

Histological sections of hepatic tissue stained with haematoxylin and eosin (H&E) are shown in Figure 5. Histopathological examination of liver sections obtained from normal control group showed normal hepatic architecture with central vein (CV) and radiating cords of normal hepatocytes (H) with central rounded vesicular nuclei and prominent nucleoli. Hepatic cords are separated by blood sinusoids (S) lined with endothelium and von Kupffer cells (blue arrow).

On the other hand, liver sections obtained from ferrous sulphate group showed dilated congested central vein (CV) with congested blood sinusoids (S). Massive fatty infiltration of hepatocytes (H) with some hepatocytes acquiring signet ring appearance (blue arrow) can also be seen in Figure 5(B).

Livers of rats treated with *N*-acetylcysteine plus ferrous sulphate showed congested central vein (CV). Normal hepatocytes (H) are separated by slightly dilated congested blood sinusoids (S) with activated von Kupffer cells (blue arrow). Binucleated cells (black arrow) can also be seen in Figure 5(C).

Livers of rats treated with saponin plus ferrous sulphate showed congested central vein (CV) and hepatocytes (H) with mild fatty deposits separated by slightly dilated congested blood sinusoids (S) and activated von Kupffer cells (blue arrow). Binucleated cells (black arrow) can also be seen in Figure 5(D).

### Effect of 10 days daily oral administration of *N*-acetylcysteine and saponin on inducible nitric oxide synthase (iNOS) and endothelial nitric oxide synthase (eNOS) expression level by immunohistochemical staining in rats with ferrous sulphate-induced hepatotoxicity

#### Inducible nitric oxide synthase (iNOS)

**Figure 6.**
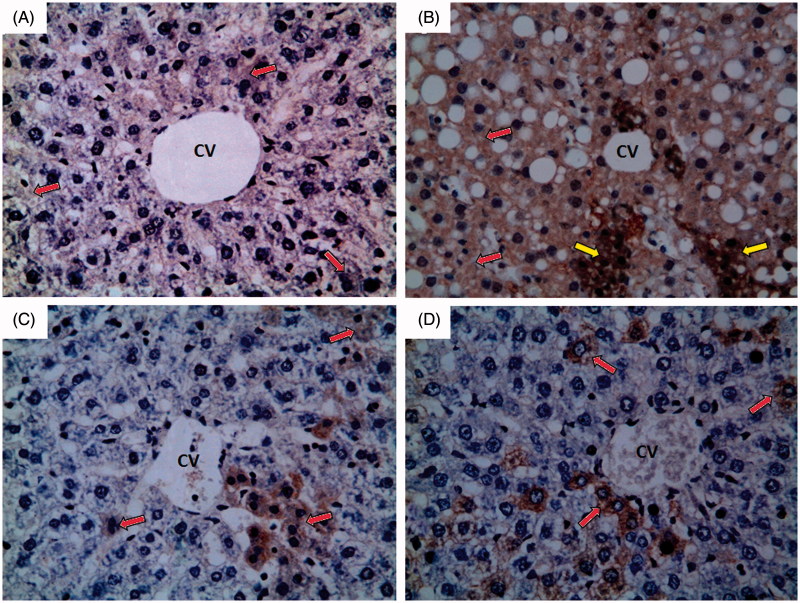
Photomicrographs of liver sections obtained from different groups (iNOS immunohistochemical stain; 400×), where (A) normal control group showing weak immunoreactivity to iNOS which appears as faint brown colour (red arrow); (B) ferrous sulphate group showing strong immunoreactivity to iNOS which appears as brown colour (red arrow) with some areas of intense immunoreactivity (yellow arrow); (C) *N*-acetylcysteine plus ferrous sulphate group showing mild immunoreactivity to iNOS which appears as brown colour (red arrow); (D) saponin plus ferrous sulphate group showing moderate immunoreactivity to iNOS which appears as brown colour (red arrow).

Normal control group showed weak immunoreactivity to iNOS which appears as faint brown colour (red arrow) as shown in Figure 6(A). Ferrous sulphate group showed strong immunoreactivity to iNOS which appears as brown colour (red arrow) with some areas of intense immunoreactivity (yellow arrow) as shown in Figure 6(B). *N*-Acetylcysteine plus ferrous sulphate group showed mild immunoreactivity to iNOS which appears as brown colour (red arrow) as shown in Figure 6(C). Saponin plus ferrous sulphate group showed moderate immunoreactivity to iNOS which appears as brown colour (red arrow) as shown in Figure 6(D).

#### Endothelial nitric oxide synthase (eNOS)

**Figure 7.**
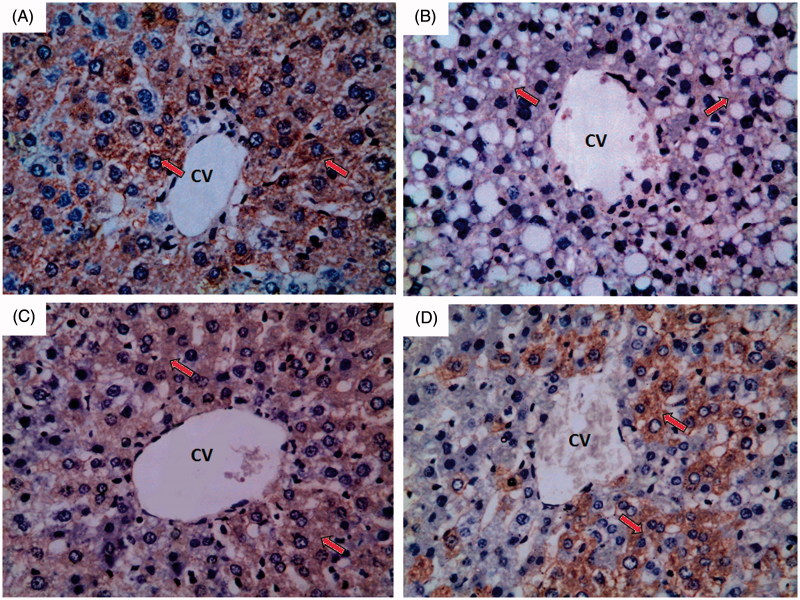
A photomicrograph of liver section obtained from different groups (eNOS immunohistochemical stain; 400×), where (A) normal control group showing strong immunoreactivity to eNOS which appears as brown colour (red arrow); (B) ferrous sulphate group showing weak to negative immunoreactivity to eNOS which appears as brown colour (red arrow); (C) *N*-acetylcysteine plus ferrous sulphate group showing strong immunoreactivity to eNOS which appears as brown colour (red arrow) but not as normal control group; (D) saponin plus ferrous sulphate group showing moderate immunoreactivity to eNOS which appears as brown colour (red arrow).

Normal control group showed strong immunoreactivity to eNOS which appears as brown colour (red arrow) as shown in Figure 7(A). Ferrous sulphate group showed weak to negative immunoreactivity to eNOS which appears as brown colour (red arrow) as shown in Figure 7(B). *N*-Acetylcysteine plus ferrous sulphate group showed strong immunoreactivity to eNOS which appears as brown colour (red arrow) but not as normal control group as shown in Figure 7(C). Saponin plus ferrous sulphate group showed moderate immunoreactivity to eNOS which appears as brown colour (red arrow) as shown in Figure 7(D).

## Discussion

Liver disease is the fifth most common cause of morbidity and mortality worldwide and the most common disease in Egypt due to the spread of hepatitis C (Cuadros et al. 2014). Liver fibrosis and liver cirrhosis are the most dangerous complications of liver injury and may cause hepatocellular carcinoma in 82% of the cases (West and Aithal 2014).

Hepatotoxicity is a common finding in iron-overloaded patients. The deposition of iron in hepatic cells produces fibrosis and cirrhosis (Weintraub et al. 1985). The present study aimed to evaluate the possible hepatoprotective effects of saponin from *Quillaja* bark as compared to the standard treatment, *N*-acetylcysteine (NAC), on acute liver injury induced by ferrous sulphate in adult male albino rats.

Iron overload-induced cell damage is believed to induce lipid peroxidation as iron is a common cofactor with the oxygen in lipid peroxidation of biological membranes (Aust et al. 1985; Bonkovsky 1991; Valko et al. 2005). The alterations in hepatocellular structure and function caused by iron overload seem to be related to free radical-mediated damage. The chemical structure of iron and its ability to drive one-electron reactions makes it a major player in the production of free radicals in the biological systems (Fraga and Oteiza 2002). Iron overload causes oxidative mitochondrial membrane damage and damage of enzymes of the tricarboxylic acid cycle (Kohgo et al. 2008). Iron has redox properties and consequently catalyzes a number of functions in living cells (Dixon and Stockwell 2014). However, these redox properties render iron able to generate ROS and destroy liver cells (Halliwell and Gutteridge 1990, 1992). Iron overload induces nitric oxide synthase expression leading to increased nitric oxide production which forms peroxynitrite through combination with superoxide anions, which is a dangerous mediator of lipid peroxidation (Chen et al. 1998, 2001).

Results of the present study showed that ferrous sulphate significantly elevated serum activities of ALT, AST, ALP, GGT and LDH indicating loss of hepatocyte membrane integrity. In agreement, Bhattacharya et al. (2000a, 2000b) stated that 30 mg/kg ferrous sulphate significantly produced hepatotoxicity in rats evidenced by significant elevations in serum ALT, AST and LDH activities, while Pari et al. (2015) stated that 30 mg/kg ferrous sulphate significantly increased serum ALT, AST, ALP, GGT and LDH activities. Serum ALT and AST levels are reliable markers for hepatocyte injury in different models (Messiha and Abo-Youssef 2015; Mohammed et al. 2016; Ali et al. 2016a). Increased serum level of ALP is due to increased synthesis in the presence of increasing biliary pressure (Manokaran et al. 2008). LDH is an intracellular enzyme, which also indicates cell membrane damage (Gaskill et al. 2005). Serum ALT, AST and LDH activities are generally increased secondary to cellular necrosis (Gaskill et al. 2005). Serum GGT has been used as indicator of liver dysfunction. Recent studies showed that serum GGT might be useful in oxidative stress estimation. In the presence of iron, the products of the GGT reaction may themselves increase the production of free radicals (Drozdz et al. 1998; Whitfield 2001; Lee et al. 2004).

Iron-induced hepatotoxicity is associated with oxidative and inflammatory events. Results of the present study revealed that ferrous sulphate caused significant increase in liver MDA and NO_x_ production as compared to normal control group, coupled with a significant reduction in liver GSH content. This comes in accordance with the results obtained by other investigators (Das et al. 2015; Pari et al. 2015).

Regarding the effect on lipid profile in the current study, ferrous sulphate administration showed significant elevation of serum levels of TG and TC. Similar results were obtained by Pari et al. (2015) who reported that serum cholesterol and triglycerides levels were significantly higher in rats after administration of 30 mg/kg ferrous sulphate. Iron overload is believed to cause disturbances of mitochondrial function, which may lead to inhibition of β-oxidation and accumulation of serum-free fatty acids and triglycerides. Serum cholesterol is also increased due to changes in the expression of the gene of the liver enzyme HMG-CoA reductase (Kojima et al. 2004).

Our results revealed that iron overload significantly reduced serum albumin level as compared to normal control group. This comes also in accordance with the results obtained by Kaur et al. (2006) who reported that serum albumin level was significantly lower in mice after i.p. administration of 9 mg/kg ferric nitrilotriacetate when compared to normal animals. Albumin is the most important protein synthesized in the liver and its concentration is a good indicator of liver synthetic capability (Hoekstra et al. 2013).

Data obtained in the present study revealed that iron overload significantly increased serum bilirubin level as compared to normal control group. This result comes in accordance with that obtained by Pari et al. (2015) who reported that serum bilirubin level was significantly higher in rats after administration of 30 mg/kg ferrous sulphate when compared to normal animals. Bilirubin is the excretory end product of heme degradation. It is conjugated in the liver with glucuronic acid and then excreted into the bile. Elevated plasma concentration of bilirubin is a marker for serious liver injury and, therefore, loss of liver function (Hoekstra et al. 2013).

The standard hepatoprotective agent *N*-acetylcysteine is one of the most potent antioxidants, being demonstrated to act as a direct antioxidant as it binds to oxygen-free radicals and serves as a cysteine donor for GSH synthesis (Bernard et al. 1984). *N*-Acetylcysteine inhibits lipid peroxidation and decreases membrane permeability resulting from any oxidant injury in the liver (Fukuzawa et al. 1995). *N*-Acetylcysteine also modulates NO production through modulation of NO synthase expression and NF-kB activity (Majano et al. 2004).

Our data showed that *N*-Acetylcysteine significantly suppressed ferrous sulphate-induced increase in serum activities of ALT, AST, ALP, GGT and LDH. This matches with the findings obtained by Shaikh et al. (1999) and Kaya et al. (2008) who stated that *N*-acetylcysteine protected liver cells against cyclosporine A-induced or cadmium-induced hepatotoxicity in rats. *N*-Acetylcysteine restored the changes in serum activities of ALT, AST, ALP, GGT and LDH due to its antioxidant effect and its ability to act as free radical scavenger, thereby protecting membrane permeability (Kaya et al. 2008).

Results of the present study also revealed that *N*-acetylcysteine caused significant decrease in liver MDA and NO_x_ production coupled with a significant increase in liver GSH content as compared to ferrous sulphate control group. *N*-Acetylcysteine showed significant decrease in serum levels of TG and TC as compared to ferrous sulphate group. Jaya et al. (1994) proved that *N*-Acetylcysteine has lipotropic activity and can reduce serum cholesterol and fatty acids. Our data also revealed that *N*-acetylcysteine significantly decreased serum bilirubin level as compared to ferrous sulphate control group. This comes in accordance with the results obtained by Ahmad et al. (2012) who reported that serum bilirubin level was significantly decreased after administration of *N*-acetylcysteine in maneb- and paraquat-induced hepatotoxicity in rats. Marked inhibition of increased levels of total bilirubin suggested that *N*-acetylcysteine offers protection against hepatotoxicity (Ahmad et al. 2012).

Accumulating evidence shows that saponins from different sources may have promising hepatoprotective effects. For instance, Jeong et al. (1997) stated that red ginseng saponins protect liver cells against carbon tetrachloride-induced hepatotoxicity in rats. Similarly, saponins obtained from *Solanum* sp. were reported to protect experimental rats against paracetamol-induced oxidative and inflammatory damage (Gupta et al. 2009). In agreement, our data showed that saponin from *Quillaja* bark significantly suppressed ferrous sulphate-induced increases in serum activities of ALT, AST, ALP, GGT and LDH. Saponin restored the changes in serum activities of ALT, AST, ALP, GGT and LDH due to its antioxidant effect, thereby protecting membrane permeability (Gupta et al. 2009).

Results of the present study revealed that saponin from *Quillaja* bark caused significant decrease in liver MDA and NO_x_ production as compared to ferrous sulphate control group coupled with a significant increase in liver GSH content. Our results are in accordance with those obtained by other investigators, where Yu et al. (2014) stated that saponin protects liver cells against carbon tetrachloride-induced hepatotoxicity in mice. Saponin is known to be an antioxidant acting by scavenging excessive radicals and suppressing oxidative stress (Lee et al. 2008; Yu et al. 2014). In a previous study, ginseng extract and total saponins inhibited LPS-induced expression of iNOS and proinflammatory cytokines in microglial cells. Furthermore, these saponins significantly suppressed MAP kinase and NF-κB activities, which are inflammatory signalling molecules (Park et al. 2009).

Concerning our results, saponin from *Quillaja* bark showed significant decrease in serum levels of TG and TC as compared to ferrous sulphate control group. This effect was possibly due to a decrease in the absorption of intestinal cholesterol and a reduction in the levels of hepatic cholesterol with increases in hepatic HMG-CoA reductase activity and LDL receptor levels in the liver (Rao and Gurfinkel 2000).

Our data revealed that saponin from *Quillaja* bark significantly decreases serum bilirubin level and significantly increases serum albumin level as compared to ferrous sulphate control group. Results of the immunohistochemical study may give another good explanation to *Quillaja* bark saponin protective activity, which is suppression of NOS expression, thus attenuating oxidative and nitrosative stress. In support, ginseng saponins could suppress iNOS activity and NO production from macrophages intoxicated with lipopolysaccharide (Jang et al. 2016). Suppression of massive NO production was previously reported to be associated with improvement of oxidative and inflammatory markers in different animal models including iatrogenic hepatotoxicity (Omar et al. 2016), bronchial asthma (Abdel-Fattah et al. 2015a, 2015b), ulcerative colitis (Ewees et al. 2016) and neurodegeneration (Ali et al. 2016b). We may conclude that *Quillaja saponaria* bark saponin is a promising hepatoprotective agent acting, at least partly, through scavenging free radicals and downregulation of nitric oxide production.
